# Relative Brain and Brain Part Sizes Provide Only Limited Evidence that Machiavellian Behaviour in Cleaner Wrasse Is Cognitively Demanding

**DOI:** 10.1371/journal.pone.0135373

**Published:** 2015-08-11

**Authors:** Dominika Chojnacka, Karin Isler, Jaroslaw Jerzy Barski, Redouan Bshary

**Affiliations:** 1 Center for Experimental Medicine, Medical University of Silesia, 40-752, Katowice, Poland; 2 Department of Zoology, University of Wroclaw, 50-335, Wroclaw, Poland; 3 Anthropological Institute and Museum, University of Zurich, 8057, Zurich, Switzerland; 4 Department of Physiology, Medical University of Silesia, 40-752, Katowice, Poland; 5 Institut de Biologie, Universite de Neuchâtel, 2000, Neuchâtel, Switzerland; University of Lethbridge, CANADA

## Abstract

It is currently widely accepted that the complexity of a species’ social life is a major determinant of its brain complexity, as predicted by the social brain hypothesis. However, it remains a challenge to explain what social complexity exactly is and what the best corresponding measures of brain anatomy are. Absolute and relative size of the brain and of the neocortex have often been used as a proxy to predict cognitive performance. Here, we apply the logic of the social brain hypothesis to marine cleaning mutualism involving the genus *Labroides*. These wrasses remove ectoparasites from ‘client’ reef fish. Conflict occurs as wrasse prefer client mucus over ectoparasites, where mucus feeding constitutes cheating. As a result of this conflict, cleaner wrasse show remarkable Machiavellian-like behaviour. Using own data as well as available data from the literature, we investigated whether the general brain anatomy of *Labroides* provides any indication that their Machiavellian behaviour is associated with a more complex brain. Neither data set provided evidence for an increased encephalisation index compared to other wrasse species. Published data on relative sizes of brain parts in 25 species of the order Perciformes suggests that only the diencephalon is relatively enlarged in *Labroides dimidiatus*. This part contains various nuclei of the social decision making network. In conclusion, gross brain anatomy yields little evidence for the hypothesis that strategic behaviour in cleaning selects for larger brains, while future research should focus on more detailed aspects like the sizes of specific nuclei as well as their cryoarchitectonic structure and connectivity.

## Introduction

There is enormous variation among vertebrate species concerning both absolute brain size and brain size relative to body size [1]. The most widely accepted explanation for variation in mammals is known as the Machiavellian intelligence [2] or social brain hypothesis [3]. While originally developed for mammals it is increasingly applied to other vertebrate clades. The basic argument as put forward by [4] is as follows. The social environment is less predictable than the physical environment, and hence needs continuous assessment. The more complex the social environment is the more challenging it becomes to keep track of the changes. Therefore, brain evolution reflects the degree of complexity of the social environment for any given species. This line of argument has been developed further by [2], with a focus on the variety of potential socio-cognitive adaptations through which an individual may exploit the potential benefits of its social world, as well as dealing with the hostile aspects of it. Such adaptations are for example perspective-taking and knowledge of social hierarchy, social play, social curiosity, social learning and teaching, and thus cultural transmission, as well as socially influenced flexibility, problem solving and innovation. The term ‘Machiavellian intelligence’ refers to the possible use of the various cognitive adaptations in the context of coalition formation against third parties as well as for the manipulation and deception of others.

On the anatomical level, empirical evidence comes from comparative data on a variety of vertebrate taxa that document positive correlations between brain size / brain structure and a species’ social life [5–17]. However, the putative indicators of social complexity vary greatly between studies, like the size of the social network [5], the strength of social bonds [6–9], the stability of groups [9, 10], the possibility for social learning [11] or habitat complexity, where habitat complexity is linked to the complexity of interspecific social interactions [12–17].

The importance of interspecific social interactions has mainly been proposed for fishes. Indeed while there is increasing evidence that at least some fish species are able to perform complex intraspecific social behaviour [18–26], some of the potentially most complex social interactions in fishes hitherto observed occur between partner species, like the case of some grouper species using functionally referential gestures during coordinated hunts with other predators like moray eels, Napoleon wrasses and octopus [27,28]. In addition, various studies identified co-evolutionary arms races between predator and prey fish as correlates of encephalization indices [29–32].

The apparently important role of interspecific interaction for brain evolution in fishes provides the basis for our current study, which focusses on the question whether analyses of the brain of cleaner wrasse of the genus *Labroides* provide any indication for increased cognitive abilities in this genus compared to other species of the same family and/or the same order. This question arises due to the many publications that suggest that potentially advanced cognition occurs in these cleaners, which remove ectoparasites but also mucus, tissue and scales from so-called client fish [33]. In particular the interactions between cleaner wrasses *Labroides dimidiatus* and their client reef fishes have various ingredients for Machiavellian-like intelligence [34] as initially proposed for primates by [2]. Cleaners recognize clients individually, they remember past interactions with clients, they may cooperate, cheat, manipulate, reconcile, produce signals out of context, and use predatory clients as social tools against aggressive clients [34]. They also adjust levels of cooperation to the presence of image scoring bystander clients [35], specific characteristics of clients like mucus quality and escape abilities [36], and the presence of a partner cleaner during inspection [37–39]. Furthermore, they remember when they interacted with whom [40]. Comparative studies that include facultatively cleaning wrasse species and cleaning gobies show that the behavioural and strategic repertoire of these other species is much simpler [41–42]. In comparative learning experiments, *L*. *dimidiatus* outperforms chimpanzees, orangutans, capuchins as well closely related wrasse in tasks designed to replicate problems that are ecologically relevant for *L*. *dimidiatus* [36,43]. Based on all these studies, we decided to investigate whether apparently complex cleaning interactions correlate with relative brain size and/or relative brain part sizes.

In a first step we studied the relationship between body size and brain size in the wrasse family. We provide two separate analyses, one based on own data and one based on published data. Our own data offer a comparison of *L*. *dimidiatus* to five other wrasse species. The other species include the closely related bicolor cleaner wrasse *Labroides bicolor*, which is also an obligate cleaner. While it has shown some evidence of potentially advanced cognition [44,45] it differs from *L*. *dimidiatus* in that individuals rove over large areas, precluding or reducing the need of several cognitive features like individual recognition, remembrance of past interactions, or paying attention to image scoring bystanders. *L*. *bicolor* individuals are less cooperative than *L*. *dimidiatus* individuals [45,46]. None of the other studied wrasse species cleans. The second analysis involved data on brain size and body size of wrasse species that are freely accessible at fishbase.org, published by [47]. While the data set comprises a large variety of clades, we focussed on wrasses in order to reduce potential variation due to independent evolutionary history between clades. Based on the behavioural data we had clear predictions concerning relative brain size. For our own samples we predicted that if strategic behaviour during cleaning interactions is cognitively demanding and this would be reflected in overall relative brain size, then *L*. *dimidiatus* should have a relatively larger brain than *L*. *bicolor*, which should have a larger relative brain than the other species. With respect to the published large data set on wrasse body and brain weights, we predicted that the brain sizes of *Labroides* species are clearly above the regression line that links brain size to body size.

In a second step we used published 140 data on the sizes of fish brain parts [48] to explore the relative sizes of various brain parts: the % of weight of telencephalon, diencephalon, mesencephalon, cerebellum and myelencephalon in relation to the entire brain. This analysis was motivated by the argument put forward by various authors that the size of specific brain areas relative to the rest of the brain would yield more valuable results about variation in cognitive performance than the relationship between overall brain size and body size [49–52]. For mammals, the neocortex ratio, i.e. the size of the neocortex relative to the rest of the brain, is often used in comparative studies [53]. It is unclear whether there could be a fish equivalent of such a ratio. We therefore considered two ratios. First, we acknowledged research on fishes suggests that telencephalon, mesencephalon and cerebellum all play a role in cognitive decision making and may form a functional integrity considered as a base in cognitive functions [54–57]. Second, we combined telencephalon and diencephalon because these two areas contain most nuclei of the so-called social decision-making network [58,59]. This network is apparently highly conserved among vertebrates and consists of the ‘social behaviour network’ and the ‘basal forebrain reward system’ [58,59]. A conserved array of hormones, neurohormones, enzymes and receptors helps to regulate key social behaviors such as parental care, aggression, mating and sexual behaviors, response to social stressors, and communication [18, 59–65]. Thus, we explore the relative contribution of each brain area as well as different combinations of brain areas to overall brain size. The published data set comprises *L*. *dimidiatus* but only one other species of the wrasse family. Therefore, we analysed all available information for the 25 species of the order Perciformes to which the wrasse family belongs [48]. Our basic prediction is that if cleaning interactions of cleaner wrasse are cognitively demanding then brain parts linked to cognitive function should be relatively larger relative to other Perciformes.

## Methods

### Ethics statement

Local Ethical Committee for Experiments on Animals, Wroclaw, Poland approved the experiment. According to permit no. 70/05 individuals intended for the study were euthanized with MS-222, dose over 250mg/l of water. Because MS-222 is a low-pH agent, its solution was buffered with sodium hydroxide to prevent shock and minimize suffering in fish transferred to it. The name of the aquarium fish import company that provided me with the fish is ReefShop BONAFIDE ul.Morska 65A, 81–323 Gdynia, Poland.

Our own data on total brain volume and body mass were collected on six fish species: the orangeback fairy-wrasse *Cirrhilabrus aurantidorsalis* (Allen and Kuiter, 1999), the cleaner wrasse *Labroides dimidiatus* (Cuvier and Valenciennes, 1839), the bicolor cleaner wrasse *Labroides bicolor* (Fowler & Bean, 1928), the birdwrasse *Gomphosus varius* (Lacépède, 1801), the canary wrasse *Halichoeres chrysus* (Randall, 1981) and the Indian pinstripe wrasse *Halichoeres vrolikii* (Bleeker, 1855) all from the Labridae family. For the interpretation of results 3 to 5 individuals from each species were used. *Halichoeres* is phylogenetically closely related to *Labroides* while *Cirrhilabrus* is very distant within the labrid family.

All species chosen for the study live in coral reefs, at depths of 1 to 30 m, in pairs or small groups. They have similar reproductive behaviour, and are often protogenic hermaphrodites. However, they differ in terms of foraging behaviour. The cleaner wrasse and bicolor cleaner wrasse are an obligatory cleaners and feeds exclusively on fish parasites. *C*. *aurantidorsalis*, *H*. *chrysus* and *H*. *vrolikii* and *G*. *varius* feed on invertebrates or small crustaceans.

All individuals used in the study were obtained from an importer of aquarium fish. We used both fish that for various causes did not survive transport or acclimatisation (16 individuals), and live, that we euthanized with MS222 (6 individuals). All appeared sexually matured in size. All individuals were fixed in formalin before weighting and dissection. A total of 22 brains was dissected. Before brain dissection, all fish were measured for morphometric features, i.e. body length and weight. The brain was dissected from the spinal cord at the level of the first spinal nerves and weighed after the removal of cerebro-spinal meninges.

Statistical analyses were done in JMP Version 11.0 (SAS Institute Inc., 2013). To compare the relative brain sizes of the six species, an ANOVA was run using brain mass as the response and body mass as the effect variable. Differences between taxa were investigated using Tukey-Kramer post hoc tests. An interactions effect for species X body mass (allowing for different slopes of the brain vs. body regression lines in the species) was not included in the model due to the very low sample size in some taxa.

For the analysis of the data available on fishbase.org, we first identified all wrasse species. The strength of the data base is that it includes a great variety of fish species. The weak points of the data base are that age/sex classes are mixed without being specified, sample sizes are highly variable and may often include only one or two specimen for a given species, and there is no information on the history of each specimen, i.e. where it was caught and whether it lived in an aquarium before sampled. The latter may affect body size in unpredictable ways. We inspected individual data points and omitted all data of juvenile fish. Data extraction was handled by RB, who used published data on maximal body length and own experience with coral reef dwelling species to decide on the minimal length of adults for each species. The remaining individual data points were then used to calculate species means for the statistical analyses.

**Fig 1:**
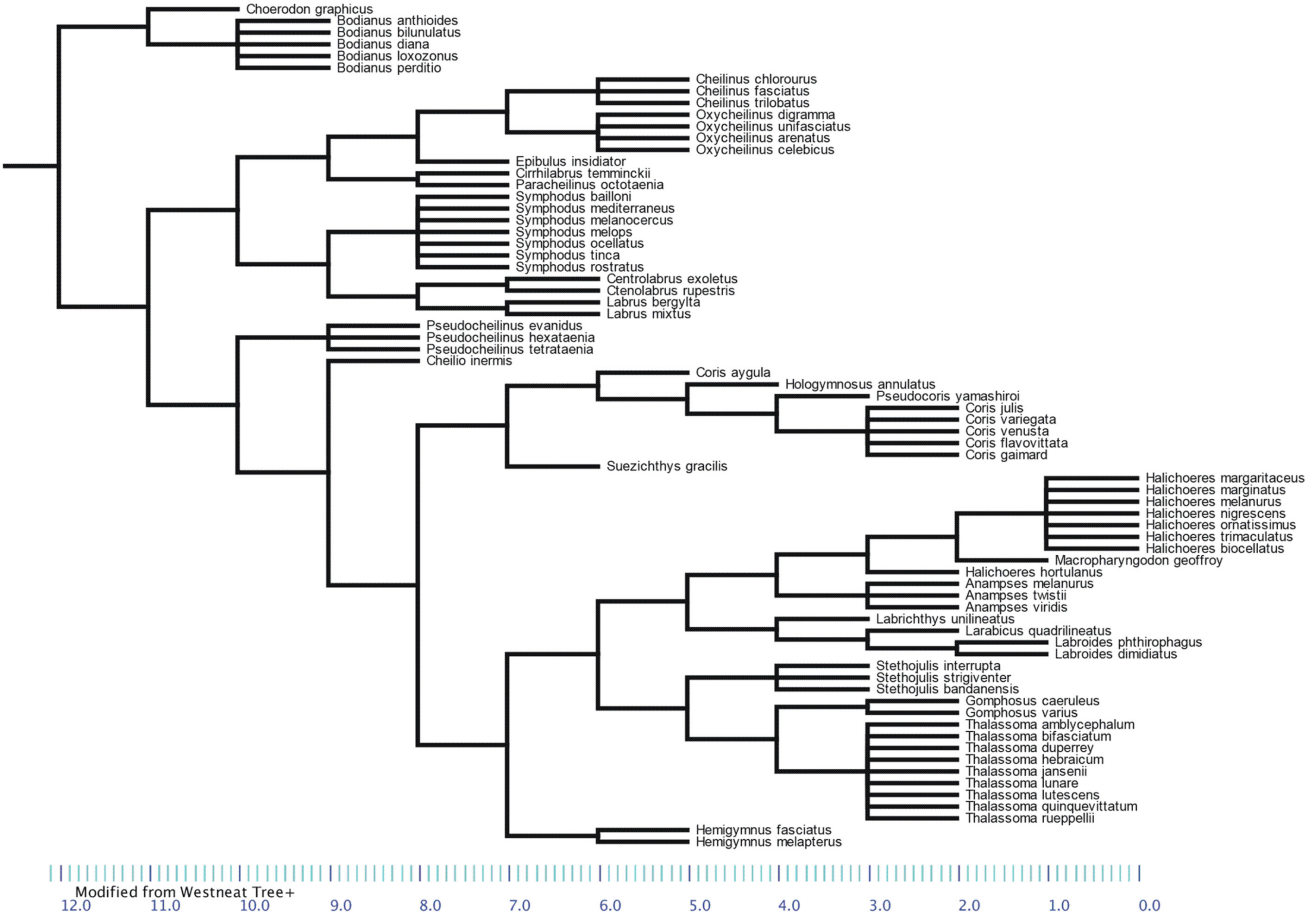
Phylogenetic tree of wrasses (N = 72 species), based on Westneat and Alfaro (2005).

In total, data on brain and body mass of N = 73 wrasse species were available on fishbase.org. For 46 of those species, a measure of total body length was also available. A phylogenetic tree for 72 of those was constructed based on Westneat and Alfaro (2005) [66] (Fig 1). *Stethojulius balteata* was excluded because no phylogenetic information was available for this genus. *Symphodus* was placed as a sister taxon to the other Labrinae (*Centrolabus*, *Ctenolabrus* and *Labrus*). As no branch length information was available, we determined the most appropriate transformation to ultrametricize the tree correlating the absolute values of the standardized contrasts of ln brain size vs. the square root of the sum of the corrected branch lengths in PDAP/PDTree [67] in Mesquite 2.75 [68]. Nee’s transformation yielded a non-significant correlation between the two, and was therefore preferred over other transformations such as Grafen’s or Pagel’s.

Phylogenetic statistical analyses were done in R, using the package caper [69]. To take phylogenetic dependencies into account, phylogenetic least-squares regressions (PGLS) were performed, using brain mass as the response and body mass or total body length as the effect variable. Brain and body mass and total body length were logarithmized before analysis to obtain normal distributions. The parameter lambda was estimated by maximum likelihood from the model residuals, indicating the amount of phylogenetic structure in the data [70]. A lambda close to 1 indicates a large amount of phylogenetic structure, while a lambda close to zero indicates no phylogenetic structure.

We also controlled for general location: most species live on coral reefs in the Indopacific, but there were also specimen from the Mediterranean, Atlantic, and Northern Sea. The difference between temperate (1) and tropical (0) locations was tested using phylogenetic ANCOVA in PGLS.

The data on relative brain part sizes are published by [48]. We extracted the data on Perciformes and ranked *L*. *dimidiatus* for each brain part of interest.

## Results

### Brain mass relative to body mass in the measured specimens

**Fig 2:**
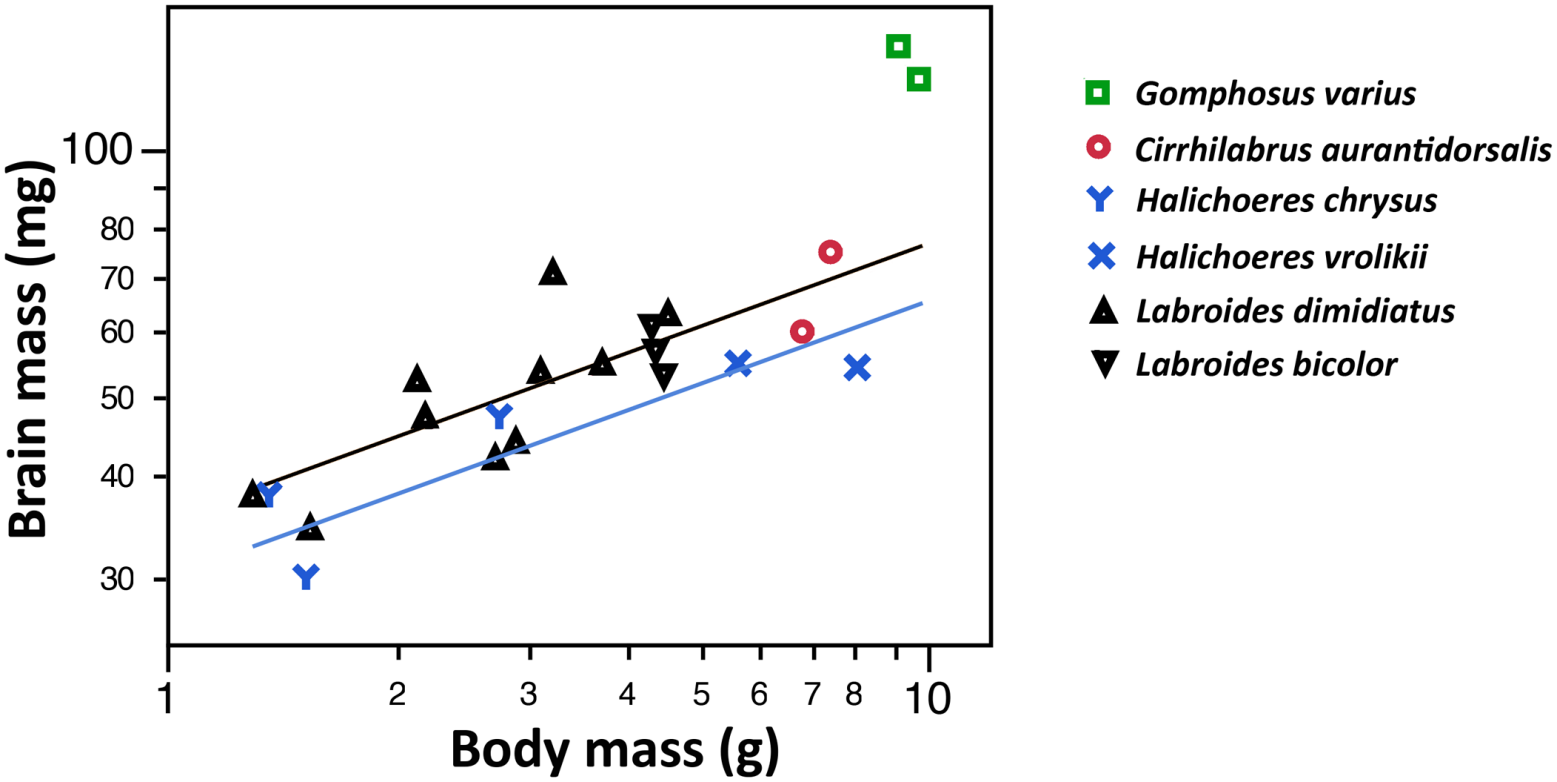
Brain mass vs. body mass (on a logarithmic scale) in 22 wrasse specimens.

Fig 2 shows the bivariate plot of logarithmized brain mass vs. body mass values in the measured wrasse specimens (N = 22 specimens from 6 species). Overall, the slope of the brain vs. body mass regression was estimated as 0.338±0.061, and the species did significantly differ (ANOVA, p(species) = 0.0004). *Gomphosus varius* individuals had a relative larger brain than the other species (ANOVA, PostHoc Tukey HSD p<0.01). *Halichoeres* was the genus with the relatively smallest brains but the difference to species other than *Gomphosus* was not statistically significant in a PostHoc test.

### Phylogenetic analysis of published data on brain vs. body mass in Labridae

The PGLS regression of ln(brain mass) vs. ln(body mass) in N = 72 Labridae species of the fishbase.org sample yields a relatively high amount of phylogenetic structure, with lambda = 0.963 being significantly different from zero, but not from 1 (p[lambda = 1] = 0.843, p[lambda = 0] = 0.0002). The slope of the phylogenetic regression is 0.556, and the correlation coefficient is r^2^ = 0.943, with an AICc of -36.90. Including location (temperate vs. tropical) as a covariate does not increase the fit of the model (p-value of location = 0.21, AICc = -36.23).

A PGLS regression of ln(brain mass) vs. ln(total body length) in N = 46 Labridae species yields qualitatively similar results, although with a higher amount of variation in brain size for any given body length (slope = 0.799, r^2^ = 0.239, AICc = 88.4, lambda = 1). Again, adding location does not improve the fit of the model (p-value of location = 0.952, AICc = 90.7)

**Fig 3:**
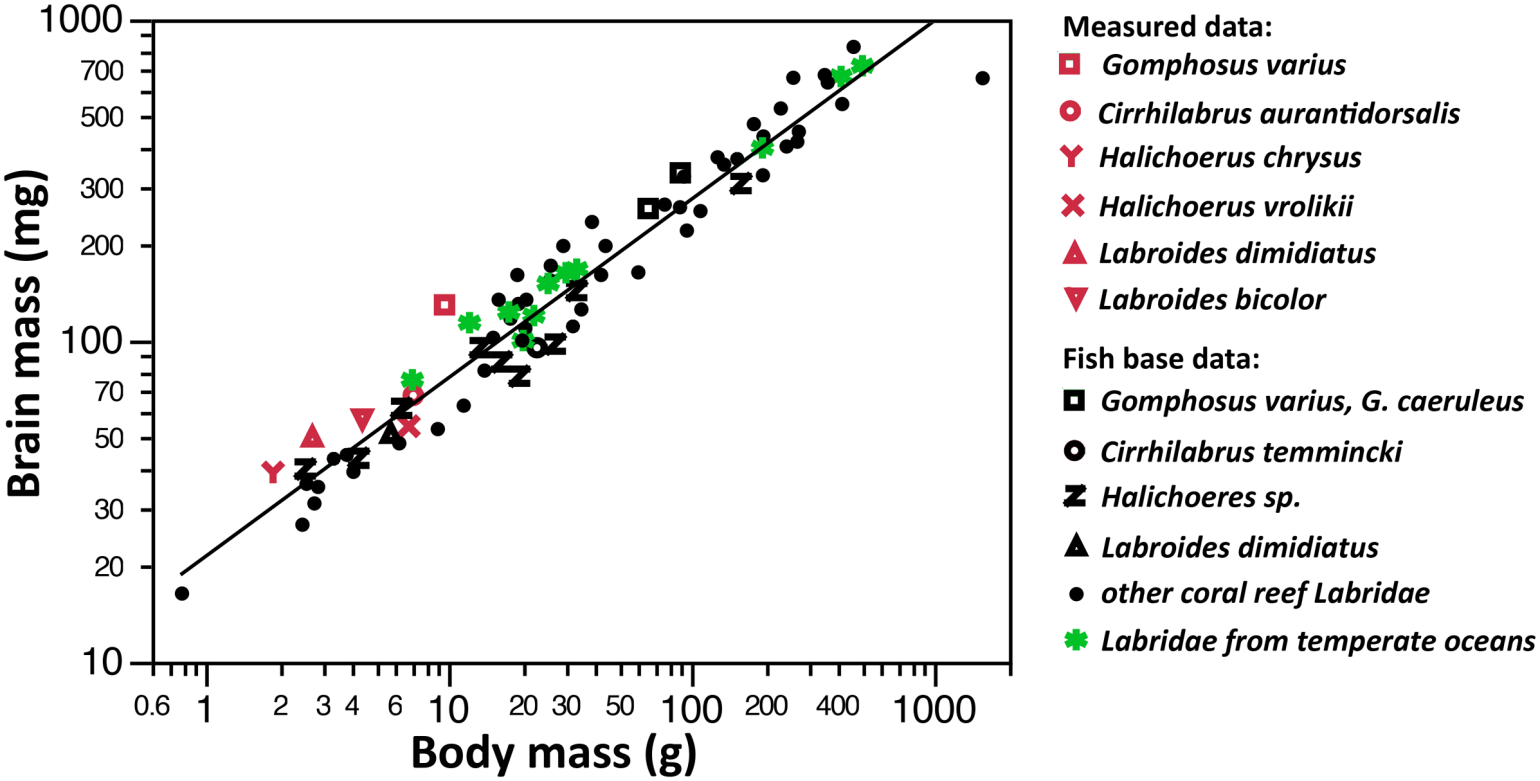
Brain mass vs. body mass (on a logarithmic scale) in 77 wrasse species. The regression line is fitted to fish base values by using a fixed slope of 0.557 from the phylogenetic least-squares regression. Species living in the Mediterranean, Atlantic, and Northern Sea are shown in green.

In Fig 3, the phylogenetic regression line is fitted into the bivariate plot of the fishbase.org species means. The plot also shows the species means of the measured specimens (in red). In both samples, *Gomphosus* had a relatively large brain for its body mass, while the *Labroides* species were more or less on the regression line, exhibiting a mean brain mass as expected for their body mass.

### Relative sizes of brain parts in *L*. *dimidiatus* compared to species of the same order Perciformes

**Fig 4:**
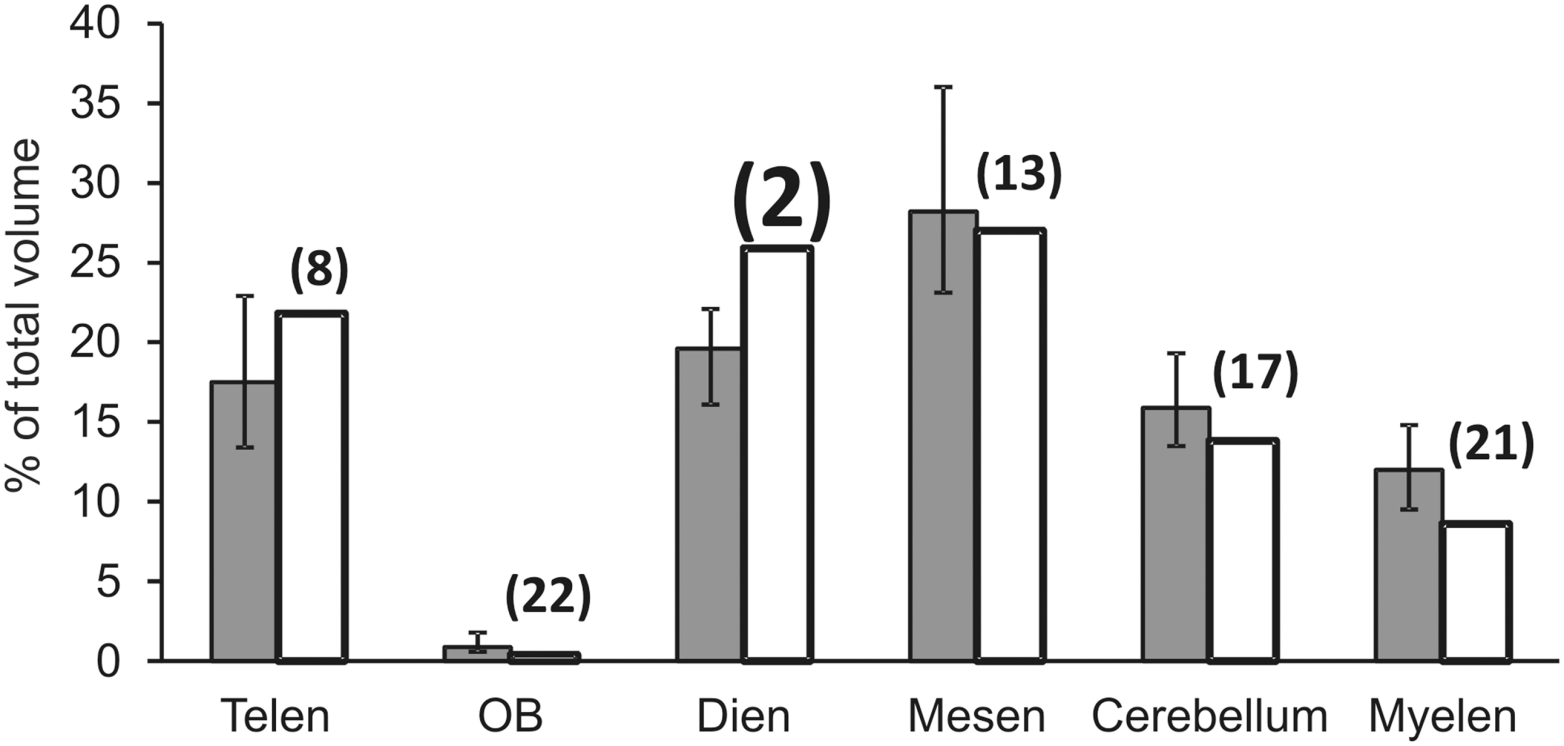
The relative size of various brain parts relative to total brain volume in *Labroides dimidiatus* (white bars) compared to 24 other species belonging to the same order Perciformes (grey bars represent median values with interquartiles). Numbers in brackets above the bars for *L*. *dimidiatus* indicate their ranking in size relative to the 24 other species. Telen: Telencephalon; OB: Olfactive Bulbs (part of the telencephalon); Dien: Diencephalon; Mesen: Mesencephalon; Myelen: Myelencephalon (pons and medulla).

The ranks of the relative size of brain parts in *L*. *dimidiatus* are typically within the interquartile range within the order Perciformes (Fig 4). This holds for the telencephalon (8^th^ out of 25 species), the mesencephalon (13^th^ out of 25) and for the cerebellum (17^th^ out of 25). The olfactory bulb and the myelencephalon (i.e. pons and medulla) are relatively small (22^nd^ and 21^st^ out of 25). Only the diencephalon is relatively large though not the largest within the order (2^nd^ out of 25). Neither the combination of telencephalon, diencephalon and cerebellum relative to the rest of the brain nor the combination of telencephalon and diencephalon relative to the rest of the brain is exceptionally large relative to other perciform species (6^th^, respectively 4^th^ out of 25).

## Discussion

We had asked whether apparently sophisticated strategies during cleaning interactions are correlated with an increase in relative brain size within the wrasse family and/or with an increase of specific brain areas within the order of perciformes. The main result of putative relevance is that *L*. *dimidiatus* has a large diencephalon relative to the rest of the brain compared to other perciformes. Indeed, several interesting brain areas involved in decision making are found in the diencephalon. The dorsal area is the location of the lateral habenula, which is activated by aversive stimuli or inappropriate outcomes and in turn affects motor and cognitive behaviors by inhibiting the activity of mesencephalic dopaminergic and serotonergic neurons [71]. Emotional assessments of interactions with clients could be very helpful in optimizing cleaning behavior (service quality, use of tactile stimulation). Furthermore, various nuclei of the social decision making network are located in the diencephalon. The social decision-making network is apparently highly conserved (i.e., putatively homologous) among vertebrates [62] and regulates social interactions. It comprises nuclei from the social behavior network and the basal forebrain reward system [58,59]. Nuclei from the social behavior network located in the diencephalon are the preoptic area, the ventral tuberal nucleus and the anterior tuberal nucleus, while the posterior tuberculum belongs to the basal forebrain reward system. In conclusion the diencephalon plays an important role for decision making in the context of social behaviour, and hence its relatively large size in *L*. *dimidiatus* may well be linked to cognitive demands inherent in cleaning interactions.

Our own data set and the data extracted from fishbase.org are consistent in suggesting that the relative brain size of *Labroides* species is not particularly large compared to other wrasse species, which has recently been described in an independent study [72]. In our own data set, *G*. *varius* appeared to have the relatively largest brains. In the online data set, the *Labroides* species brain sizes were very close to the sizes predicted by the regression line. We hence tentatively conclude that cleaning does not select for relatively larger brains. In addition, as discussed above it appears that the only candidate for the enlargement of a specific brain area in *L*. *dimidiatus* is the diencephalon. In contrast, the relative sizes of the telencephalon, the mesencephalon and the cerebellum seem to be quite average, at least in comparison to other species of the same order.

Somewhat unexpectedly, we also found no effect of location on relative brain sizes. Species living in the Mediterranean, Atlantic, and Northern Sea had similar encephalisation indices compared to the indopacific coral reef dwelling species. As previous studies concluded that the complexity of the interspecific social network correlates positively with brain size [12,16,17,73,74] wrasse species from the Indopacific should have had the relatively largest brains, given that these species live in the most fish species-rich area of the world. Apparently, more detailed information about the ecology of each species would be highly valuable in order to test hypotheses about the effects of social interactions that go beyond cleaning.

### Methodological considerations

All data sets consist of typically few individuals per species. Unfortunately, the variance in both our data and the data on fishbase.org is considerable. Thus, it remains a possibility that our negative results might be driven by low sample sizes.

With respect to overall brain weights, there were systematic differences between the two data sets: our own data yielded higher encephalisation indices than the online data set. It seems possible that our fish had all lost weight during the transport to Poland, as fishes are not fed in that time period. The online data base does not specify for each specimen whether it was wild caught and killed on site or treated differently. As we do not know why the data sets yield such different results, we conclude that different data sets should not be mixed for analyses. Any future samples should be very specific about processing of specimen, so that hopefully a more reliable data base can be built, where forms of processing and their effects as well as location and time of year can be included in the analyses.

### Are cleaning interactions not cognitively demanding?

Our data on brain sizes relative to body mass and on the relative size of specific brain areas mostly yield the conclusion that on such basic anatomical levels the brain of cleaning wrasse species and of *L*. *dimidiatus* in particular are organized in similar ways as closely related non cleaning species. A possible exception is the size of the diencephalon relative to the rest of the brain in *L*. *dimidiatus*. When we discuss these findings, it is important to consider two potential explanations. First, that cleaning interactions are not that cognitively demanding, second that cleaner wrasse have adjusted to the cognitive challenges by a reorganization of the brain rather than by increasing its size. Both ideas have their potential merits.

How could it be possible that cleaner wrasse show sophisticated social decision rules without a corresponding larger brain? A possible answer is that the sophisticated behaviours are all due to operant conditioning, facilitated by the fact that cleaners may have 2000 interactions per day [75]. Thus, cleaners obtain feedback from clients about the consequences of their own behavior 2000 times a day. As long as cleaners are able to identify few relevant stimuli—client categories like predator, resident, visitor, presence of bystanders, joint inspection—they thus have ample opportunities to associate with each stimulus what level of service quality is optimal, without the need for an overall larger brain. According to data published by [76], adult cleaners are faced with the decision to choose between a resident and a visitor client about 2.3 times per hour, hence 25 times in an 11h day, and 750 times per month. As adult cleaners normally have a clear preference for visitors in the wild [77] it might well be that their performance in laboratory market experiments using Plexiglas plates that is superior to primates reflects ontogenetic experience rather than evolved cognitive adaptations [43]. The same line of argument may apply to their ability to feed against preference [36]. Finally, [78] recently repeated experiments mimicking biological market [79] or social prestige problems [80] on cleaners from marginal habitats harbouring low competition over access to clients and low client diversity. These cleaners have only about 800 interactions per day but spend about as much time cleaning because interactions last longer [78]. The cleaners were largely unable to solve the tasks, which suggests an important role of ontogeny for the expression of sophisticated decision rules. Nevertheless, we note that colleagues have proposed that the cerebellum plays a major role in operant conditioning [81,82], and hence one might have expected that this brain part was enlarged in *L*. *dimidiatus*. This was not the case, however, in comparison to other species of the same order. Comparisons between more closely related species might have been more informative as they may indicate recent adjustments to trade-offs. Indeed, a very recent study compared relative brain part sizes in two *Labroides* species and two species from closely related genera (*Labropsis* and *Labrichthys*) describes that *L*. *dimidiatus* has relatively larger cerebellum and brainstem [72]. Thus, these data support the hypothesis that *L*. *dimidiatus* might be particularly adapted to associative learning, while our data do not. The relative brain part sizes of *L*. *bicolor* are invariably average in those recent analyses [72].

Fish brain size and functioning is quite flexible both on ecological and evolutionary time scales. Fish brains are highly plastic, and variation can be linked to cognitive performance [83,84]. [85] showed that relative brain size diverged within 1–2 generations in response to artificial selection in guppies (*Poecilia reticulata*). However, it is conceivable that cleaner wrasse cannot easily alter the size of their brains in response to selection on increased cognitive performance because there should also be selection on small head sizes in cleaners for two reasons. First, the mouth needs to remain small to allow cleaners to be efficient in removing small ectoparasites from the clients’ surface. A comparative analysis of labrid jaw anatomy found that cleaners have a small mouth, small muscles, low jaw protrusion, low maxillary KT and high jaw-opening mechanical advantage [86]. In addition to jaw anatomy, it appears to us from observations of cleaning interactions that cleaners detect prey with stereo-type vision as they feed on items in front of them. This feeding mode suggests a very close focal point for stereo-view that enables them to find often cryptic prey on the clients’ surface. Enlargement of the brain would lead to a greater distance between eyes, which might reduce efficiency in finding food.

If cleaner wrasse are constrained with respect to brain size evolution, selection on increased cognitive abilities could lead to a restructuring of their brain instead of an enlargement. For example, it has been argued that marine mammals like cetaceans do not face major constraints on head size evolution and hence may evolve large brains with relatively low cell density, at least in comparison to primates [87]. The bird song system provides a clear example that size is not always a function of cognitive ability, i.e. song complexity and repertoire size. While there is a general positive correlation between repertoire size and HVC size across species [88]. Relations between song repertoire size and the volume of brain nuclei related to song: comparative evolutionary analyses amongst oscine birds, males and females in duetting song bird species that sing the same repertoire differ significantly in HVC size (males have a larger HVC than females do) [89].

## Conclusions/Outlook

Our results strongly suggest that cleaner wrasse do not have particularly enlarged brains relative to closely related species. We discussed two potential explanations, namely that the apparently complex decision rules about service quality are either not particularly cognitively demanding but the result of long exposure to operant conditioning opportunities, or that cleaners responded to selection for increased cognitive abilities with a restructuration of the brain rather than with an increase in brain size. Thus, the next step will be to look at the cleaners’ brain architecture. Particularly promising areas are the nuclei involved in the social decision making network, which consists of the ‘social behaviour network’ and the ‘basal forebrain reward system’ [58,59]. Until we know about the cleaners’ brain structure, it remains an open question in how far their Machiavellian-like behaviour needs particular cognitive adaptations [44].
